# Genomic characterization of ceftazidime/avibactam-resistant KPC-producing *Klebsiella pneumoniae* in bloodstream infections indicates OmpK35 and OmpK36 as pivotal key players in resistance mechanisms

**DOI:** 10.3389/fmicb.2026.1840520

**Published:** 2026-06-15

**Authors:** Claudia Vaiana, Roberta Vazzana, Michela Gagliardo, Claudia Carcione, Elena Conoscenti, Stijn Blot, Francesco Monaco, Francesca Cardinale, Daniele Di Carlo, Alessandra Mularoni, Valentina Agnese, Pier Giulio Conaldi, Nicola Cuscino, Alessia Gallo

**Affiliations:** 1 IRCCS ISMETT, Palermo, Italy; 2 Fondazione Ri.MED, Palermo, Italy; 3 UPMC Italy, Palermo, Italy; 4 Department of Internal Medicine and Pediatrics, Ghent University, Ghent, Belgium

**Keywords:** antimicrobial resistance, antimicrobial stewardship, bloodstream infections, *K. pneumoniae*, whole genome sequencing

## Abstract

**Background:**

The clinical management of bloodstream infections (BSIs) caused by KPC-*Klebsiella pneumoniae* has been severely challenged by the emergence of ceftazidime/avibactam (CAZ/AVI) resistance. The circulation of CAZ/AVI-resistant KPC-Kp remains a public health critical concern, warranting the need of strict epidemiological surveillance and genomic analysis to elucidate the underlying molecular mechanisms.

**Methods:**

We retrospectively investigated 16 BSIs caused by KPC-Kp isolates resistant to CAZ/AVI collected from January 2022 to July 2025. Antimicrobial susceptibility testing (AST) was performed to assess resistance profiles against last-line agents. Whole Genome Sequencing (WGS) and bioinformatic analyses were employed to identify molecular drivers of resistance and evaluate genetic relatedness. Patients’ data were collected to integrate clinical outcomes with the microbiological features of the isolated strains.

**Results:**

The BSIs analyzed were characterized by a high degree of severity, with 9/16 patients presenting a Pitt bacteremia score ≥ 4 and 7/16 (44%) a 30-days mortality rate. Intestinal colonization by KPC-Kp was identified in 14/16 patients, and prior CAZ/AVI-based therapy had been administered to 12/16, suggesting the resistance development under selective antimicrobial pressure. AST revealed resistance to last-line agents: imipenem/relebactam (4/16), meropenem/vaborbactam (2/16) and colistin (3/16), alongside reduced susceptibility to cefiderocol (11/16), thereby limiting effective treatment strategies. Besides the predominance of high-risk clones, such as ST101 (8/16) and ST512 (3/16), phylogenetic analysis highlighted a cluster within ST101 group, suggesting a hospital outbreak. Furthermore, two novel sequence types, ST9507 and ST8640 were identified. Multiple KPC variants were detected, including the novel KPC-293 and KPC-296, which were not previously described. All isolates exhibited a truncated OmpK35 in combination or not with a highly mutated OmpK36, with insertion in loop3 being the most prevalent alteration. Given the heterogeneity of the KPC variants found in the isolates, and the consistent loss of porin function, the OmpK35/36 impairment may represent the pivotal driver of the MDR phenotype in these strains.

**Conclusion:**

Our findings indicate that the synergy between multiple KPC variants and porin structural alterations may represent a critical determinant underlying the clinical challenge posed by CAZ/AVI resistance in invasive infections.

## 1 Introduction

The global spread of carbapenem-resistant Enterobacterales (CRE) has emerged as one of the most critical public health threats of the 21st century, substantiated by a significant increase in prevalence documented over recent years (Ma et al., 2023). Within this class of drug-resistant pathogens, *Klebsiella pneumoniae* strains harboring the *Klebsiella pneumoniae* carbapenemase (KPC), commonly referred as KPC-Kp, represent a critical challenge in clinical environments. Considering their high incidence, transmissibility rates, the complex resistance patterns and unfavorable clinical outcome, KPC-Kp has recently been included in the “critical priority” group of the WHO Bacterial Priority List 2024 (WHO Bacterial Priority Pathogens List, 2024)^1^. Bloodstream infections (BSIs) caused by KPC-Kp are characterized by alarming high mortality rates, thus necessitating urgent and targeted clinical interventions (Bassetti et al., 2018; Giacobbe et al., 2023b). KPC enzymes remain largely unaffected by conventional beta-lactamase inhibitors such as clavulanic acid, tazobactam, and sulbactam (Hobson et al., 2022). Furthermore, these strains frequently co-harbor resistance determinants against other major antibiotic classes, including aminoglycosides, quinolones, sulfonamides, and tetracyclines, further narrowing the available therapeutic window (Li et al., 2023). The therapeutic approaches for KPC-Kp infections have undergone a significant transformation over the last decade, following the introduction of novel beta-lactam/beta-lactamase inhibitor (BL/BLI) combinations. Among these, ceftazidime/avibactam (CAZ/AVI) serves as the primary therapeutic choice, while meropenem/vaborbactam (MER/VAB) and imipenem/relebactam (IMI/REL) are used as last-line antibiotics (Gaibani et al., 2022; Giacobbe et al., 2023a). Moreover, recent studies have pointed out that the increase of mortality rates in carbapenem-resistant Kp BSI is not dependent only on therapeutic strategy but other clinical prognostic factors may be involved (Giacobbe et al., 2023b).

The dissemination of high-risk clones like ST512, which is endemic in Southern Italy, alongside ST101 and ST307, which have been found to be responsible for BSIs, highlights the critical need for a rigorous epidemiological surveillance and continuous monitoring within the hospital settings (Loconsole et al., 2020). In Italy, a transition from the sporadic resistance to more diffuse horizontal transmission has been observed, with alarming hospital outbreaks in Rome and Turin. These events were characterized by rapid transmission speeds (17 cases in 60 days in Turin) and high mortality rates (nearly 24% in Rome). Interestingly, in both cases, the resistance was driven by specific mutations within the *bla*KPC gene (Boattini et al., 2026; Campogiani et al., 2023). Resistance to CAZ/AVI is a complex, multifactorial phenomenon, often involving alterations at multiple molecular levels. Some of the most common mutations are located within the *bla*KPC gene and are associated with their over-expression or the appearance of novel KPC subtypes (KPC-2 and KPC-3) and variants (KPC-31 and KPC-33) (Bianco et al., 2026; Boattini et al., 2024b). In addition to this, it has been shown that resistance is frequently mediated by changes in membrane permeability caused by mutations or loss of expression of outer membrane porins like OmpK35 and OmpK36 (Bianco et al., 2026; Boattini et al., 2024b; Wang et al., 2020). Given that CAZ/AVI resistance has become a global healthcare concern (Di Bella et al., 2021; Gonçalves et al., 2025), a comprehensive understanding of the molecular mechanisms underlying these resistance patterns remains essential for implementing future antimicrobial strategies and for an effective infection control. The primary aim of this study was to perform a detailed genomic characterization of bacterial strains isolated from BSIs in critically ill patients. In particular, we focused on the identification of specific *bla*KPC variants and on the porin mutation pattern that drive CAZ/AVI resistance. Moreover, we aimed to correlate these genomic findings with the clinical course, considering patient demographics, treatment history, and 30-days mortality in order to better evaluate the impact of these highly resistant strains on the clinical outcome. The integration of genomic and clinical data may not only guide local empirical therapy but also strengthen global surveillance efforts against emerging antimicrobial threats.

## 2 Materials and methods

### 2.1 Clinical data collection and bacterial strains isolation

During the study period from January 2022 to July 2025 a total of 117 patients, admitted at IRCCS-ISMETT (Mediterranean Institute for Transplantation and Advanced Specialized Therapies) hospital in Palermo, Italy, were found to have blood cultures positive for KPC-Kp. BSI was defined according to the standard criteria requiring at least a single positive peripherally sampled blood culture together with compatible clinical signs. Among the 117 patients, 30 (25.64%) had BSIs caused by KPC-Kp isolates resistant to CAZ/AVI. In particular, 12 cases occurred in 2022, 8 in 2023, 6 in 2024, and 4 by July 2025. Only 16, out of the 30 patients, were included in the present study, due to the availability of the bacterial isolates. Enrolled patients were followed up until death or for 30 days from the date of infection. A retrospective approach was utilized to collect patient demographic and clinical data from hospital electronic records. The collected data included reason for admission, BSI severity based on Pitt bacteremia score (Al-Hasan and Baddour, 2020; Papadimitriou-Olivgeris et al., 2021), risk factors for multidrug resistance (i.e., prior hospitalization and surgery, prior antibiotic exposure, solid organ transplant recipient or other relevant immunosuppression, intestinal colonization with Carbapenemase-producing Enterobacteriaceae (CPE), central venous access, chronic dialysis intended as maintenance dialysis prior to hospital admission/infection onset), antibiotic treatment, and clinical outcomes (i.e., length of hospital stay, 30-days mortality from the index culture). Exposure data were collected according to the previously described working definition of treatment-emergent resistance, which captures phenotypic resistance occurring during therapy or within a defined follow-up period of 180 days (Falcone et al., 2026). Antibiotic therapy was considered appropriate when at least one antibiotic agent was administered with *in vitro* and likely clinical activity against the isolated strain.

Bacterial identification and antimicrobial susceptibility testing were performed by the hospital diagnostic laboratory as part of the routine clinical management. Subsequently, these bacterial isolates were stored at −80°C in Luria-Bertani (LB) broth supplemented with 20% glycerol for genomic analysis.

### 2.2 Bacterial identification and antimicrobial susceptibility testing

Blood cultures (BCs) were performed using the BacT/ALERT Virtuo detection system with FA and FN Plus bottles (bioMérieux, Marcy l’Ètoile, France). Positive BCs were sub-cultured on the appropriate solid media. The bacterial species were identified using Matrix-Assisted Laser Desorption Ionization Time-of-Flight Mass Spectrometry (MALDI-TOF MS) with the Vitek^®^ MS system (bioMérieux, Marcy l’Étoile, France). Antimicrobial susceptibility testing (AST) was performed using the VITEK-2 system (bioMérieux, Marcy l’Etoile, France), while resistance to CAZ/AVI (0.016–256/4 μg/mL) was assessed with *E*-test-based method (bioMérieux, Marcy-l’Étoile, France). The colistin resistance was confirmed by a broth microdilution based commercial kit (UMIC Colistin; Bruker Daltonics GmbH & Co., KG, Germany). Susceptibility to cefiderocol was tested with ComASP^®^ cefiderocol microdilution panel (0.008–128 μg/mL) (Liofilchem^®^, Roseto degli Abruzzi, Italy). Minimum inhibitory concentration (MIC) results were interpreted according to the EUCAST clinical breakpoints (EUCAST clinical breakpoint guidance v16.0). Production of Class A and B carbapenemases was phenotypically verified using carbapenemase inhibition tests with boronic acid derivatives (BA) and dipicolinic acid (DPA)/EDTA (Rosco Diagnostica A/S, Taastrup, Denmark). Molecular identification of five major carbapenemases (KPC, NDM, VIM, IMP, and OXA-48-like) was performed on bacterial isolates using the NG-Test Carba 5 immunochromatographic test (NG Biotech, Guipry, France), following the manufacturer’s instructions.

### 2.3 Whole genome sequencing and bioinformatics

Pure bacterial colonies were grown on MacConkey agar plates and then genomic DNA was extracted using the QIAmp DNA Mini kit (Qiagen, Hilden, Germany), by following manufacturer’s instructions. DNA quantity was assessed using the Qubit fluorometer 2.0 (Invitrogen, Carlsbad, CA, USA) with the Qubit dsDNA Broad Range assay kit. DNA libraries were prepared with the Illumina DNA Prep kit (Illumina, San Diego, CA, USA) and Nextera DNA CD Indexes (96 Indexes-96 Samples, Illumina, San Diego, CA, USA) according to the manufacturer’s instructions, as previously described (Vazzana et al., 2025). DNA libraries quality evaluation was performed with the Agilent 4200 TapeStation System (Agilent Technologies Ltd., USA), while libraries concentration was checked using the Qubit fluorometer 2.0 (Invitrogen, MA, USA). Whole Genome Sequencing was performed on the NextSeq550 System using a 2 × 150 bp paired-end library (Illumina^®^ Inc., San Diego, CA, USA) according to the manufacturer’s instructions, as previously shown (Vaiana et al., 2025, 2026). Assemblies of the draft genome and plasmids were performed using SPAdes v.3.15.12 software^2^. Prokka v.1.14.5^3^ was used to perform automatic annotation.

The Sequence Type (ST) was assigned using the MLST 2.0 tool provided by the Center for Genomic Epidemiology (CGE)^4^ (Larsen et al., 2012). Contigs containing KPC genes were aligned to plasmids identified using PlasmidFinder with the BLASTn algorithm (BLAST+ v.2.16.0) (Camacho et al., 2009). Antimicrobial resistance genes (ARGs) were detected using CARD (Alcock et al., 2020). All sequence data have been submitted to the National Center for Biotechnology Information (NCBI) database under BioProject PRJNA1418605. The whole-genome phylogenetic tree was constructed using Parsnp v1.2 (Harvest suite) (Treangen et al., 2014), which performs core-genome alignment and SNP-based phylogenetic inference under a maximum likelihood framework. Genomic distance relationships between isolates were further visualized as a graph-based tree using a custom R script, highlighting pairwise distances between genomes. Multiple sequence alignment of the CAA09665.1 and pdb_00006rd3 as reference protein sequences for OmpK35 and OmpK36, respectively. Graphical representations of the protein alignments were generated using a custom Python script.

### 2.4 Statistical analysis

Due to the descriptive nature of this study and the small sample size (*n* = 16), data were analyzed using descriptive statistics. Categorical variables (e.g., mortality rates, presence of resistance genes, and ST distribution) were expressed as absolute numbers and percentages. Continuous variables, such as the Pitt Bacteremia score and length of stay, were summarized using medians and ranges. Due to the study’s focus on the molecular characterization of a small, specific cohort (*n* = 16), formal inferential statistics and comparative *p*-value analyses were not performed to avoid overinterpretation. Data management and clinical summaries were performed using Microsoft Excel, while genomic visualizations, including the phylogenetic tree and protein alignment diagrams, were generated using custom R and Python scripts, respectively.

## 3 Results

### 3.1 Description of patients’ clinical features

Clinical characteristics of patients (*n* = 16) are reported in Table 1.

**TABLE 1:** Clinical features description.

Demographic and clinical features	
Gender, male (%)	13 (81)
Age, years (median [IQR])	63 [48–67]
Reason for admission	
Solid organ transplantation	5 (30)
Other thoracic/abdominal surgery	7 (44)
Persistent fever in solid transplant patient	3 (18)
Severe COVID-19 infection	1 (6)
Length of stay before BCKpn isolation, days (median [IQR])	30 [4–70]
Risk factors	
Prior hospitalization (<3 months)	15 (94)
Solid organ transplant	8 (50)
Liver	6
Liver/kidney	1
Kidney	1
Major surgery	10 (62)
Prior antibiotic exposure	16 (100)
KPC-Kp intestinal colonization	14 (88)
Central venous access	15 (94)
Chronic dialysis	6 (38)
Possible source of BCKpn-caused BSI	
Intra-abdominal infection	6 (38)
Primary/CLABSI	4 (25)
Surgical site infection	2 (12)
Respiratory tract infection	2 (12)
Urinary tract infection	1 (6)
Skin and soft tissue infection	1 (6)
Prior antibiotic exposure	
Any antibiotic class	16 (100)
BL/BLI*	15 (94)
CAZ/AVI	12 (75)
Piperacillin/tazobactam	9 (56)
MEM/VAB	5 (31)
Carbapenems	8 (50)
Aminoglycosides	11 (69)
Polymyxins	4 (25)
Third-generation cephalosporins	6 (38)
Fluoroquinolones	3 (19)
Glycopeptides	4 (25)
Penicillins	7 (44)
Pitt Bacteremia score ≥ 4 (%)	9 (56)
Pitt Bacteremia score ≥ 7 (%)	6 (38)
AAT^#^	12 (76)
Total length of stay, days (median [IQR])	58 [17–101]
30-days mortality (%)	7 (44)

Data are expressed as number, (%) or median [IQR].

*Including CAZ/AVI, MER/VAB, piperacillin/tazobactam (PIP/TAZ). ^#^Appropriate antibiotic therapy.

Overall, patients exhibited a risk profile consistent with severe infection and multidrug-resistant organism involvement, characterized by high rates of solid organ transplantation or other major surgical interventions, high Pitt Bacteremia Scores, prolonged hospitalization, intestinal colonization with KPC-Kp, chronic dialysis, and prior antibiotic exposure. All 16 patients received at least one antibiotic prior to the BSI. Exposure to multiple antibiotic classes was common: three patients were exposed to three classes, six patients to four classes, three patients to five classes, and one patient to six classes. Among the 16 patients, prior exposure to β-lactam/β-lactamase inhibitor (BL/BLI) combinations was common: CAZ/AVI in 12 (75%), piperacillin–tazobactam in 9 (56%), and MER/VAB in 5 (31.3%). Overall, 15 patients (93.8%) received at least one BL/BLI. Pitt Bacteremia Scores were ≥4 in nine patients, indicating critical illness, and ≥7 in six patients, reflecting a markedly increased risk of death. Twelve patients received appropriate antibiotic therapy, and seven patients died. A more detailed clinical data description for each patient is available in Supplementary Table 1.

### 3.2 Antimicrobial susceptibility profile of the isolates

AST results are reported in Table 2. All CAZ/AVI-resistant isolates were resistant to cephalosporins (cefepime, ceftriaxone, and ceftazidime) and fluoroquinolones (ciprofloxacin), while 11 out of 16 (68.7%) exhibited concomitant resistance to meropenem. Moreover, isolates showed resistance to other last-line therapeutic options, comprising IMI/REL (4/16, 25%), MEM/VAB (2/16, 12.5%), and cefiderocol (FDC) (4/16, 25%). However, within the cefiderocol-susceptible group, 7 isolates showed reduced susceptibility with a MIC of 2 μg/mL. Notably, BCKpn14 and BCKpn15 exhibited resistance to both carbapenems and BL/BLIs. Finally, colistin resistance was observed in 3 out of 16 isolates studied (18.75%).

**TABLE 2:** Antimicrobial susceptibility profile of the isolates analyzed.

MIC (μg/ml)																		
Id strain	Date of strain isolation	Immuno- chroma-tographic test	AMK	AMC	FEP	CTX	CAZ	CIP	GEN	IMI	MEM	PIP/ TAZ	CEF/ TAZ	CST	MEM/ VAB	IMI/ REL	CAZ/ AVI	FDC
BCKpn1	1/30/2022	KPC	32 (R)	>16 (R)	>16 (R)	>32 (R)	>32 (R)	>2 (R)	>8 (R)	>8 (R)	>8 (R)	>64 (R)	>16 (R)	0.5 (S)	2 (S)	0.5 (S)	>256 (R)	2 (S)
BCKpn2	2/11/2022	KPC	32 (R)	>16 (R)	>16 (R)	>32 (R)	>32 (R)	>2 (R)	>8 (R)	>8 (R)	>8 (R)	>64 (R)	>16 (R)	0.5 (S)	4 (S)	0.5 (S)	>256 (R)	2 (S)
BCKpn3	2/18/2022	KPC	32 (R)	>16 (R)	>16 (R)	>32 (R)	>32 (R)	>2 (R)	>8 (R)	>8 (R)	>8 (R)	>64 (R)	>16 (R)	0.5 (S)	2 (S)	2 (S)	12 (R)	2 (S)
BCKpn4	4/5/2022	KPC	ND	>16 (R)	>16 (R)	>32 (R)	>32 (R)	>2 (R)	>8 (R)	8 (R)	>8 (R)	>64 (R)	>16 (R)	0.5 (S)	4 (S)	0.5 (S)	>256 (R)	16 (R)
BCKpn5	4/18/2022	NEG	ND	>16 (R)	>16 (R)	>32 (R)	>32 (R)	>2 (R)	>8 (R)	≤0.25 (S)	2 (S)	>64 (R)	>16 (R)	16 (R)	≤0.5 (S)	≤0.25 (S)	>256 (R)	4 (R)
BCKpn6	9/9/2022	KPC	ND	>16 (R)	>16 (R)	>32 (R)	>32 (R)	>2 (R)	>8 (R)	4 (I)	>8 (R)	>64 (R)	>16 (R)	0.5 (S)	≤0.5 (S)	≤0.25 (S)	64 (R)	0.5 (S)
BCKpn7	11/4/2022	KPC	32 (R)	>16 (R)	>16 (R)	>32 (R)	>32 (R)	>2 (R)	>8 (R)	>8 (R)	>8 (R)	>64 (R)	>16 (R)	0.5 (S)	4 (S)	1 (S)	12 (R)	0.25 (S)
BCKpn8	1/5/2023	NEG	32 (R)	16 (R)	>16 (R)	>32 (R)	>32 (R)	>2 (R)	>8 (R)	≤0.25 (S)	1 (S)	64 (R)	>16 (R)	0.5 (S)	≤0.5 (S)	≤0.25 (S)	>256 (R)	2 (S)
BCKpn9	3/15/2023	KPC	4 (S)	>16 (R)	>16 (R)	>32 (R)	>32 (R)	>2 (R)	>8 (R)	≤0.25 (S)	≤0.25 (S)	>64 (R)	>16 (R)	0.5 (S)	≤0.5 (S)	0.5 (S)	>256 (R)	4 (R)
BCKpn10	8/2/2023	KPC	≤1 (S)	>16 (R)	>16 (R)	>32 (R)	>32 (R)	>2 (R)	≤1 (S)	>8 (R)	>8 (R)	>64 (R)	>16 (R)	0.5 (S)	≤0.5 (S)	≤0.25 (S)	24 (R)	0.5 (S)
BCKpn11	10/10/2023	KPC	32 (R)	>16 (R)	>16 (R)	>32 (R)	>32 (R)	>2 (R)	>8 (R)	>8 (R)	>8 (R)	>64 (R)	>16 (R)	0.5 (S)	8 (S)	4 (R)	12 (R)	0.5 (S)
BCKpn12	11/11/2023	KPC	8 (S)	>32 (R)	>16 (R)	>32 (R)	>32 (R)	>2 (R)	>8 (R)	ND	4 (I)	>64 (R)	>16 (R)	0.5 (S)	4 (S)	4 (R)	>256 (R)	2 (S)
BCKpn13	4/3/2024	NEG	ND	>32 (R)	>16 (R)	ND	>32 (R)	>2 (R)	>8 (R)	ND	>8 (R)	>64 (R)	>16 (R)	0.5 (S)	8 (S)	0.5 (S)	>256 (R)	4 (R)
BCKpn14	8/22/2024	KPC	ND	>32 (R)	>16 (R)	ND	>32 (R)	>2 (R)	>8 (R)	ND	>8 (R)	>64 (R)	>16 (R)	4 (R)	32 (R)	4 (R)	64 (R)	2 (S)
BCKpn15	2/5/2025	KPC	48 (R)	>32 (R)	>16 (R)	ND	>32 (R)	>2 (R)	128	>8 (R)	>8 (R)	>64 (R)	>16 (R)	4 (R)	32 (R)	>8 (R)	64 (R)	1 (S)
BCKpn16	4/30/2025	NEG	2 (S)	>32 (R)	>16 (R)	ND	>32 (R)	>2 (R)	>8 (R)	ND	4 (I)	>64 (R)	>16 (R)	0.5 (S)	2 (S)	0.5 (S)	>256 (R)	2 (S)

The table reports the MIC values obtained with AST. The antimicrobial agents tested were: AMC, amoxicillin/clavulanic acid; AMK, amikacin; FEP, cefepime; CTX, cefotaxime; CAZ, ceftazidime; CAZ/AVI, ceftazidime/avibactam; CEF/TAZ, ceftolozane/tazobactam; CIP, ciprofloxacin; CST, colistin; GEN, gentamicin; IMI, imipenem; IMI/REL, imipenem/relebactam; MEM, meropenem; MEM/VAB, meropenem/vaborbactam; PIP/TAZ, piperacillin/tazobactam; FDC, cefiderocol. NEG, negative. S, susceptible; R, resistant; I, intermediate; ND, not determined.

### 3.3 Molecular pattern and genetic clustering of the isolates

**FIGURE 1:**
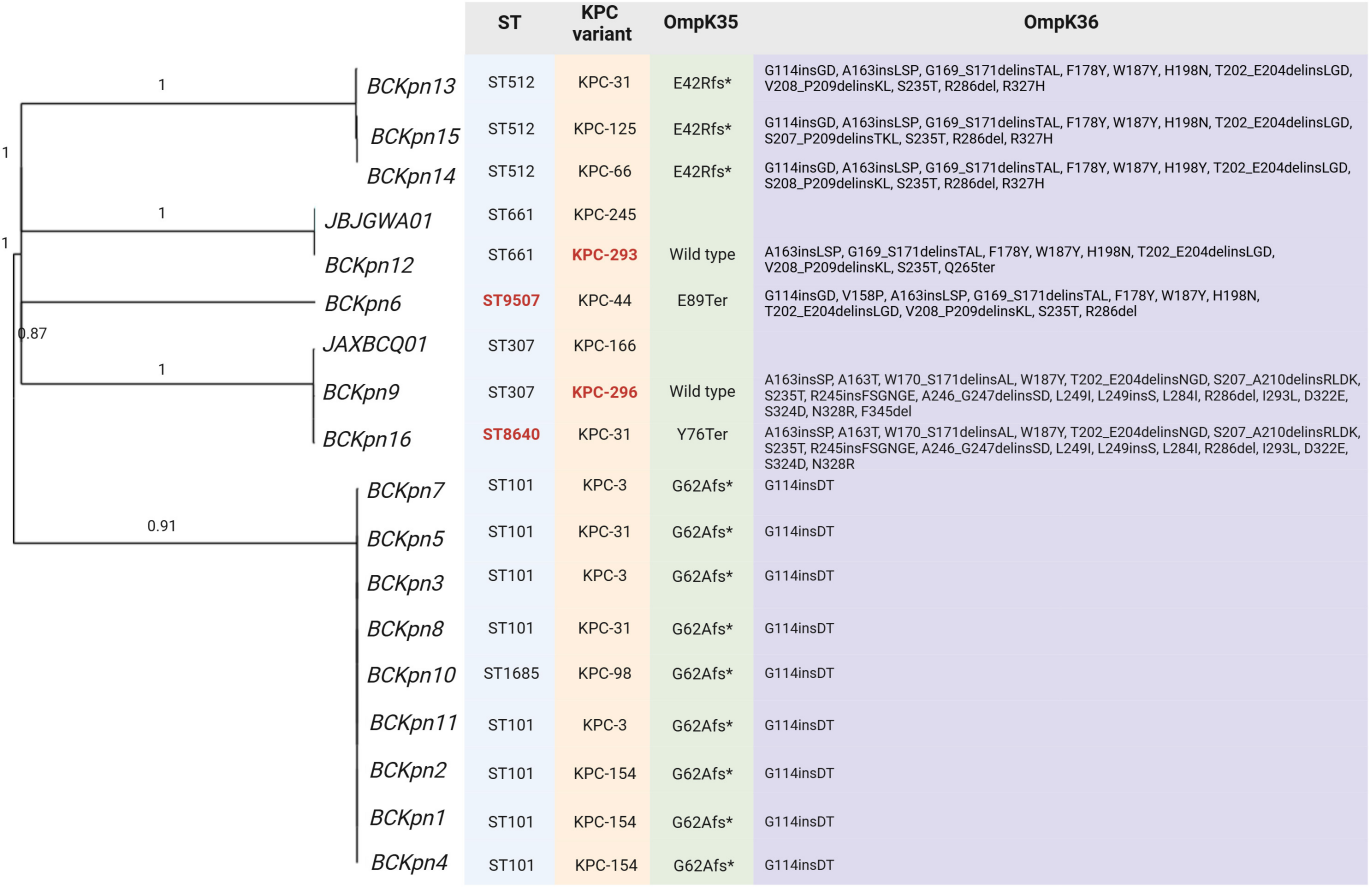
Phylogenetic and molecular profiling of KPC-Kp isolates. The dendrogram displays the genetic relationships among the clinical isolates analyzed in this study alongside two external CAZ/AVI-resistant KPC-Kp strains: JBJGWA000000000, an ISMETT circulating strain (Vaiana et al., 2025), and JAXBCQ000000000.1, associated with an Italian hospital outbreak (Piazza et al., 2024). Numbers at nodes represent SH-like local support values computed by FastTree2 (within the Parsnp pipeline) and are shown as decimal numbers (from 0 to 1). SH-like local support values are indicated only for the major internal nodes to ensure clarity; all the values for terminal branches and a high-resolution tree containing all support values are provided in Supplementary Figure 1 from left to right, the table summarizes: ST, KPC variant, mutational variations of the OmpK35 and OmpK36 porins. Specific amino acid substitutions, insertions (ins), deletions (del) and nonsense mutations (ter) are described for each isolate. Novel STs and novel KPC are highlighted in red. * Represents a premature stop codon caused by a frameshift mutation. This image was created using BioRender (https://www.biorender.com/).

The molecular profiles and phylogenetic analysis of the isolates are shown in Figure 1.

**FIGURE 2:**
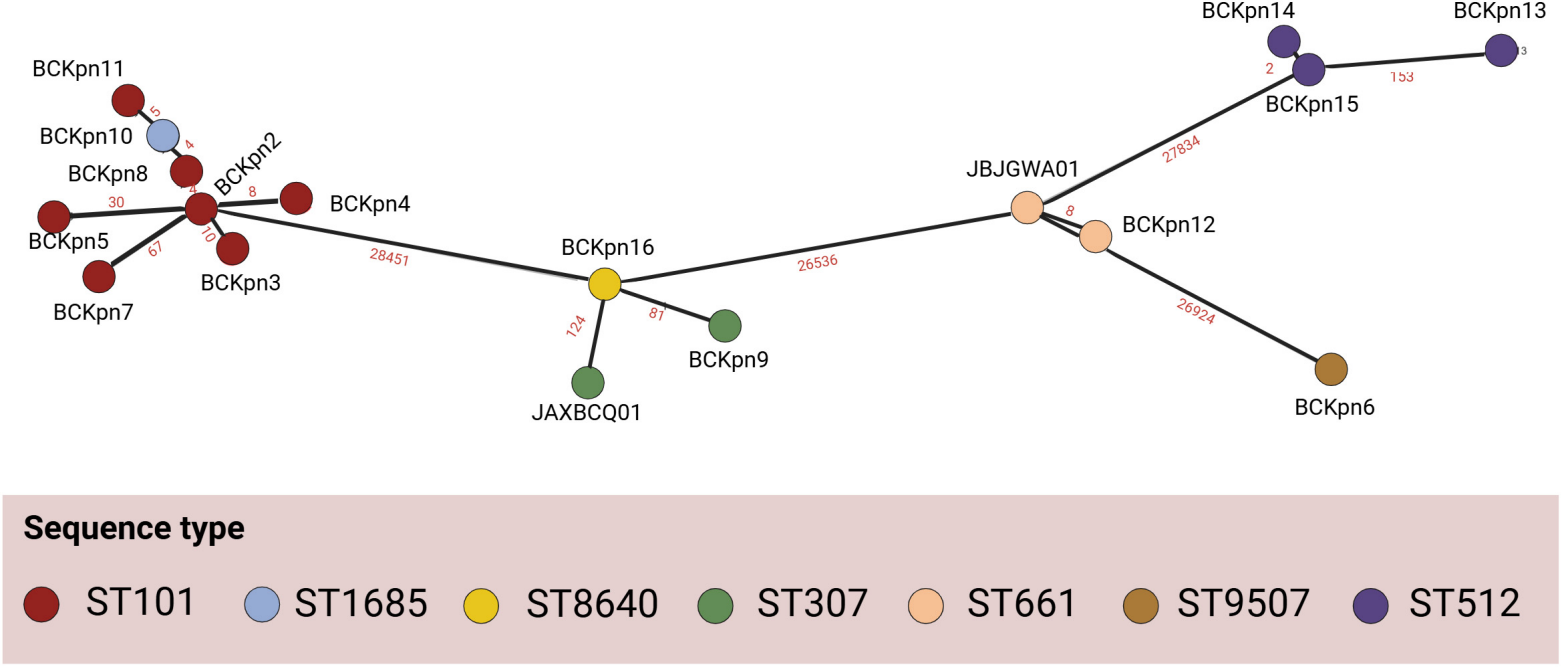
Minimum spanning tree of the isolates based on core genome SNP analysis. The tree was generated using a custom R script. In red, numbers represent pairwise SNP numbers between isolates. The color of the nodes indicates the specific ST they belong to. ST: sequence type. This image was created using BioRender (https://www.biorender.com/).

MLST analysis revealed that ST101 was the predominant sequence type, accounting for 50% (8/16) of the strains. Notably, these isolates clustered together phylogenetically (median pairwise SNP distance of 15), reflecting their shared temporal origin. Indeed, except for a single isolate collected in October 2023, all strains were collected from January 2022 to January 2023 (Figure 2).

**FIGURE 3:**
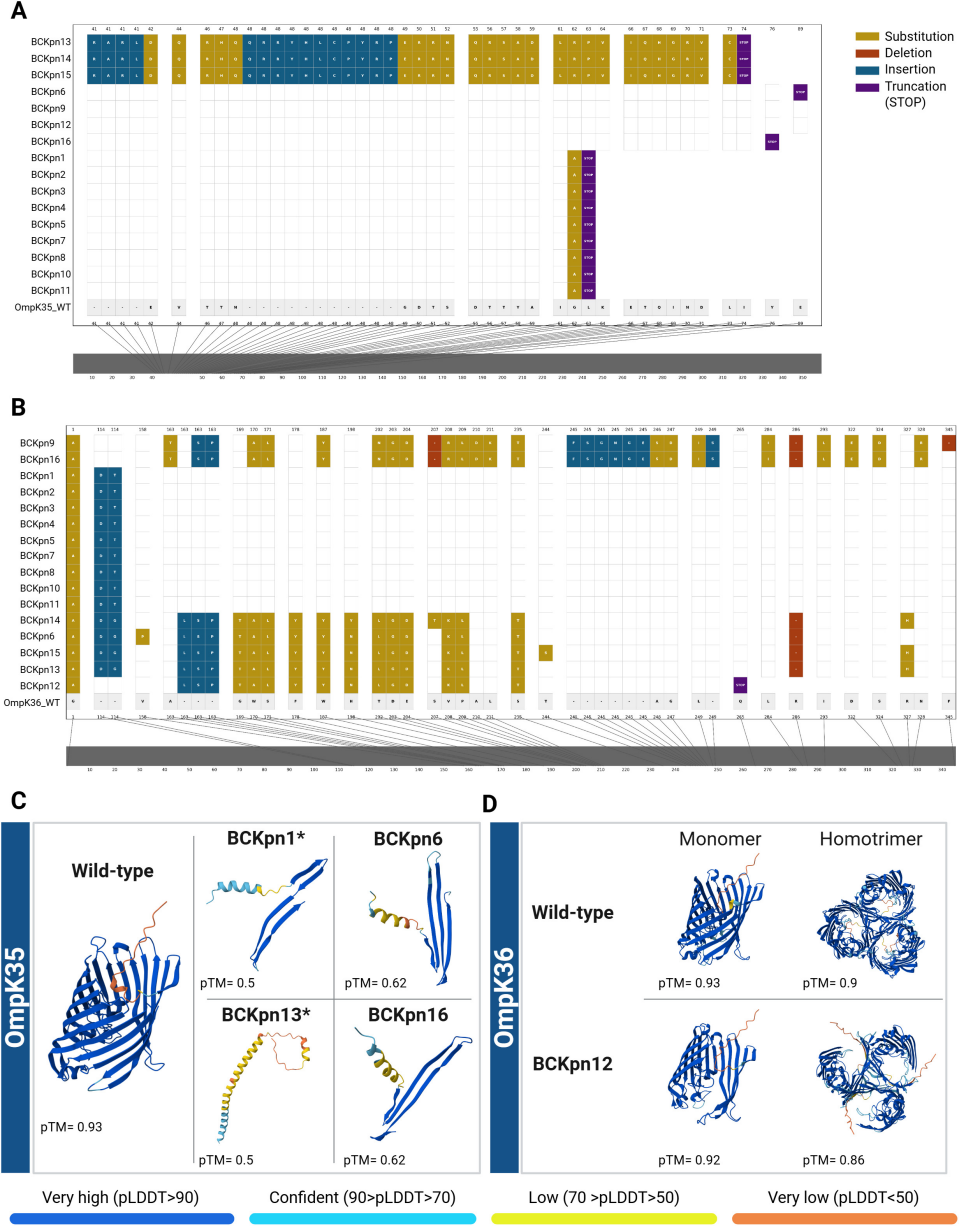
Molecular characterization and predicted 3D structures of OmpK35 and OmpK36 porins in KPC-Kp isolates. Multiple ORF sequence alignments show amino acid variations in OmpK35 **(A)** and OmpK36 **(B)** compared to the wild-type reference proteins. **(C)** Alpha-Fold3 (Abramson et al., 2024) predicted 3D protein structures of OmpK35 wild-type and mutants described in this study. Asterisks highlight the isolates that show the same OmpK35 amino acidic sequence as other isolates analyzed. In particular, BCKpn1* shows the same sequence as BCKpn2, 3, 4, 5, 7, 8, 10 and 11, and BCKpn13* with BCKpn14 and 15 (as shown in **A**). In panel **(D)**, predicted 3D protein structures of OmpK36 wild-type and BCKpn12 mutant are shown. pTM represents the predicted template modeling score of the AlphaFold3 prediction, that measures the accuracy of the entire structure (Xu and Zhang, 2010; Zhang and Skolnick, 2004). pTM scores above 0.5 corresponds to a high-confidence fold prediction. pLDDT is the predicted Local Distance Difference Test and represents the per-atom confidence estimation of the AlphaFold3 prediction. This image was created using BioRender (https://www.biorender.com/).

Among them, BCKpn1 and BCKpn2 were genetically identical (no SNPs). Beyond their temporal and genetic clustering, they exhibited significant molecular similarities, showing the *bla*KPC-3 in 3 out of 8 isolates, *bla*KPC-154 in 3 out of 8 isolates and *bla*KPC-31 in 2 out of 8 isolates variants. All ST101 isolates consistently harbored a truncated OmpK35 porin at amino acid 62 and a DT insertion in loop3 of OmpK36, as depicted in Figures 3A,B. Furthermore, ST1685-BCKpn10 exhibited the same porin mutation profile and shared high genetic similarity with the ST101 cluster.

Regarding the three ST512 strains, collected over a 6-months period, BCKpn14 and BCKpn15 were genomically indistinguishable, while BCKpn13 differed for 153 SNPs. Although carrying distinct KPC variants (KPC-66, -31, -125), they shared the same porin mutation pattern, consisting of a truncated OmpK35 protein of 88 aa (Figures 3A–C) and an OmpK36 carrying several mutations, including a DG-insertion in loop 3 (Figures 3B–D).

One isolate (BCKpn12), belonging to ST661, showed significant similarity (8 SNPs) to the ISTTKP0124 strain previously isolated and characterized in our hospital (Vaiana et al., 2025). It carried a novel KPC variant (KPC-293), showing 99.91% identity with *bla*KPC-125 (NG_080778.1); the predicted protein sequence differed by a single missense mutation (E274D) (Supplementary Table 2). Despite carrying a wild-type OmpK35, a truncated OmpK36 porin of 287 aa was found, leading to substantial structural alterations (Figure 3D).

Another KPC variant (KPC-296), carried by BCKpn9 (ST307) isolate, was here identified. It harbors a *bla*KPC variant showing the highest sequence identity (96.95%) to the *bla*KPC-41 gene (NG_065876.1) and encodes a predicted protein characterized by a 9-amino acid insertion (Ins170AIPGDARDT171), as depicted in Supplementary Table 2.

In this study, two novel sequence types, ST9507 (BCKpn6) and ST8640 (BCKpn16), were characterized. These strains exhibited no significant genetic relatedness to the other isolates and carried distinct molecular profiles characterized by the *bla*KPC-44 and *bla*KPC-31 variants, respectively. Both strains harbored truncated OmpK35 porins resulting from nonsense mutations (E89Ter and Y76Ter, respectively) alongside severely compromised OmpK36 porins. The 3D structure prediction of the OmpK35 proteins identified in our isolates confirmed that, when compared to the wild-type protein, the truncated proteins showed a non-conserved overall architecture (Figure 3C), suggesting the loss of function of the pore itself. Among the OmpK36 proteins, we found a truncated protein sequence in BCKpn12 isolate where the premature stop codon at position 265 (Figure 3B) resulted in an altered monomer architecture, as shown in Figure 3D. Importantly, our AlphaFold predictions of both OmpK35 and OmpK36 wild-types corresponded perfectly to the 3D structure of the proteins obtained by analyzing previously published crystal structures (pdb_00005o77 for OmpK35 WT; pdb_00006rd3 for OmpK36 WT). Bioinformatic analysis revealed that *bla*KPC genes in all isolates were located on distinct plasmids (Supplementary Table 3).

Whole Genome Sequencing analysis revealed the consistent presence of beta-lactamase-encoding genes, as reported in Supplementary Table 4. Various *bla*SHV alleles were present in all isolates, with *bla*SHV-1 being the most prevalent, followed by *bla*SHV-11, *bla*SHV-28, and *bla*SHV-27. Additionally, *bla*CTX-M-15 and *bla*TEM-1 were identified in 31.2% isolates each. Finally, *bla*OXA-type enzymes (specifically *bla*OXA-1 and *bla*OXA-10) were harbored by 37.5% isolates. Moreover, we detected additional genetic determinants associated with resistance to aminoglycosides (*aac*(6′)-*Ib10*, *aph*(3′′)-*Ib*, *aph* (6)-*Id*, *aadA2*, *ant*(2′′)-*Ia*), fluoroquinolones (*qnrB1* and *qnrS1*), sulfonamides (*sul1* and *sul2*) and trimethoprim (*dfrA12* and *dfrA14*) (Supplementary Table 4).

## 4 Discussion

Despite the introduction of the latest BL/BLI combinations in clinical practice offered a significant improvement in the treatment of carbapenem-resistant infections, the emergence of CAZ/AVI resistance represents a critical challenge especially when associated with systemic infections. Our study aimed to characterize the molecular mechanisms leading to CAZ/AVI resistance in KPC-Kp strains responsible for BSIs over a 4-years period (January 2022 to July 2025). We observed a remarkably high prevalence of CAZ/AVI resistance (25.64%) within our cohort, surpassing rates previously reported in other Italian studies (Boattini et al., 2024a; Gaibani et al., 2021; La Bella et al., 2022). This discrepancy likely reflects the specific clinical environment and infection dynamics of the transplant center.

Clinical data analysis revealed extreme BSI severity, with 56% of patients presenting a Pitt score ≥ 4 and a 30-days mortality rate of 44%. Intestinal colonization by KPC-Kp, known as a risk factor for BSI emergence (Colaneri et al., 2025), was observed in 88% of patients. Furthermore, 75% of patients had received a CAZ/AVI-based therapy before the isolation of the resistant strain, suggesting the treatment-emergent resistance driven by the selective pressure on resistant subpopulations (Boattini et al., 2026). It is important to note that the observed resistance patterns were interpreted in the context of prior antibiotic exposure using the working definition of treatment-emergent resistance (Gonçalves et al., 2025), which captures phenotypic resistance developed during therapy or within a defined follow-up period. Although this approach does not distinguish resistance arising *de novo* under antibiotic selective pressure from resistance acquired through cross-transmission or colonization, this aspect should be considered when evaluating clinical and epidemiological implications.

In this study, the concomitant resistance to latest BL/BLI combinations exhibited by CAZ/AVI-resistant isolates narrows further availability of effective therapeutic strategies. Comparative analysis with published data revealed that the co-resistance rate for MEM/VAB here observed (12.5%) was lower than previously documented values (Gonçalves et al., 2025; Lombardo et al., 2022). Conversely, the IMI/REL co-resistance rate (25%) aligns with one report (Lombardo et al., 2022) while remains below another (Gonçalves et al., 2025), a divergence that underscores the variability in regional antimicrobial profiles. Overall, these results suggest that the understanding of the resistance landscapes remains fragmentary, necessitating further investigation. Moreover, the reduced susceptibility to FDC, reaching 68.75%, warrants particular attention. Our data are in line with other studies reporting higher FDC resistance rates among CAZ/AVI-resistant KPC-Kp strains compared to wild-type (Bianco et al., 2022) and that the presence of specific KPC variants, conferring resistance to CAZ/AVI, may be also involved in the FDC susceptibility reduction (Poirel et al., 2022).

The genomic landscape of our study population was characterized by seven distinct STs, including two newly identified (ST9507 and ST8640). ST101 (50%) and ST512 (18.7%) emerged as the most predominant clones, consistently with national epidemiological trends (Bianco et al., 2025; De Koster et al., 2022), both of which have been associated with nosocomial outbreaks (Comandatore et al., 2013; Loconsole et al., 2020). Of note, the high genetic similarity and temporal relatedness within the ST101 cluster suggest a hospital outbreak lasting over a year, highlighting the urgent need for enhanced infection control measures. The remaining STs, including ST661, ST307 and ST1685, contributed to the epidemiological heterogeneity of the cohort and have been previously implicated in clinical outbreaks. ST307 has been reported to be globally disseminated (Peirano et al., 2020), while ST661, defined as a rare lineage, was implicated in an outbreak in the United Kingdom (Martin et al., 2017). Lastly, the two novel sequence types, ST9507 and ST8640, likely emerged through independent introduction events.

Regarding the molecular basis of resistance, previous studies reported that specific mutations in *bla*KPC gene and alterations in membrane permeability due to porin loss are implicated in CAZ/AVI resistance development (Shen et al., 2017; Wang et al., 2020). Our findings indicate that all isolates carried at least one non-functional porin, while the 87.5% (14 out of 16) harbored both OmpK35 and OmpK36 mutated, thus resulting in being functionally highly compromised. Specifically, among the several mutations observed, the most frequently detected were an amino acid insertion in loop 3 ring of OmpK36, responsible for pore constriction (David et al., 2022), and OmpK35 truncations, described to enhance the effect of resistance mutations in OmpK36 (Fajardo-Lubián et al., 2019; Wong et al., 2019).

Furthermore, it has been postulated that the mechanisms driving the co-resistance to CAZ/AVI and MEM/VAB and/or IMI/REL rely on the concomitant presence of *bla*KPC variants and porin alterations (Bianco et al., 2025; Shen et al., 2017). All isolates here described harbored several *bla*KPC variant genes, including two newly identified KPC-293 and KPC-296, and an impaired porin pattern, mainly showing a truncated OmpK35 and a GD insertion in loop 3 ring of OmpK36, in line with the latest findings published (Bianco et al., 2025). Given the substantial heterogeneity observed in *bla*KPC molecular pattern, the porin impairment, rather than the presence of specific carbapenemase variants alone, appeared to serve as a pivotal mechanism driving the MDR phenotype in these strains.

While our findings provide significant insights into the dynamics of bacterial resistance, highlighting the emergence of multiple KPC variants and porin alterations, both of which are critical determinants in the clinical challenge of CAZ/AVI resistance, some limitations must be considered. Among a total of 30 CAZ/AVI-resistant KPC-Kp strains responsible for BSI, genomic sequencing was performed on a subset of 16. The relatively small sample size reduces the statistical power, rendering the analysis primarily descriptive and the findings potentially not generalizable to a broader population.

Our study provides an exhaustive elucidation of the molecular profile of CAZ/AVI-resistant KPC-Kp responsible for BSIs, integrating comprehensive clinical data and in-depth bacterial WGS characterization. By expanding the pivotal work of Gaibani et al. (2020), our study focused on a larger cohort to more broadly explore the mechanisms and clinical implications of this emerging threat. Ultimately, our findings underscore the urgent need for enhanced genomic surveillance and tailored stewardship to mitigate the spread of MDR KPC-Kp clones.

## Data Availability

The raw Illumina WGS data are available in the NCBI SRA database with the accession number: PRJNA1418605. https://www.ncbi.nlm.nih.gov/bioproject/PRJNA1418605/.

## Footnotes

1
https://iris.who.int/handle/10665/376776

2
https://github.com/ablab/spades

3
https://github.com/tseemann/prokka

4
https://cge.food.dtu.dk/services/MLST/

## Supplementary material

Supplementary Figure 1 High-resolution phylogenetic tree of KPC-Kp isolates with complete SH-like local support values at all nodes.

Supplementary Table 1 Demographic and clinical data for each patient.
