# Supplementary figures and images for: Genomic characterization of ceftazidime/avibactam-resistant KPC-producing Klebsiella pneumoniae in bloodstream infections indicates OmpK35 and OmpK36 as pivotal key players in resistance mechanisms
Source: Front Microbiol. 2026 Jun 15;17:1840520. doi: 10.3389/fmicb.2026.1840520 (PMC13311080; doi:10.3389/fmicb.2026.1840520)

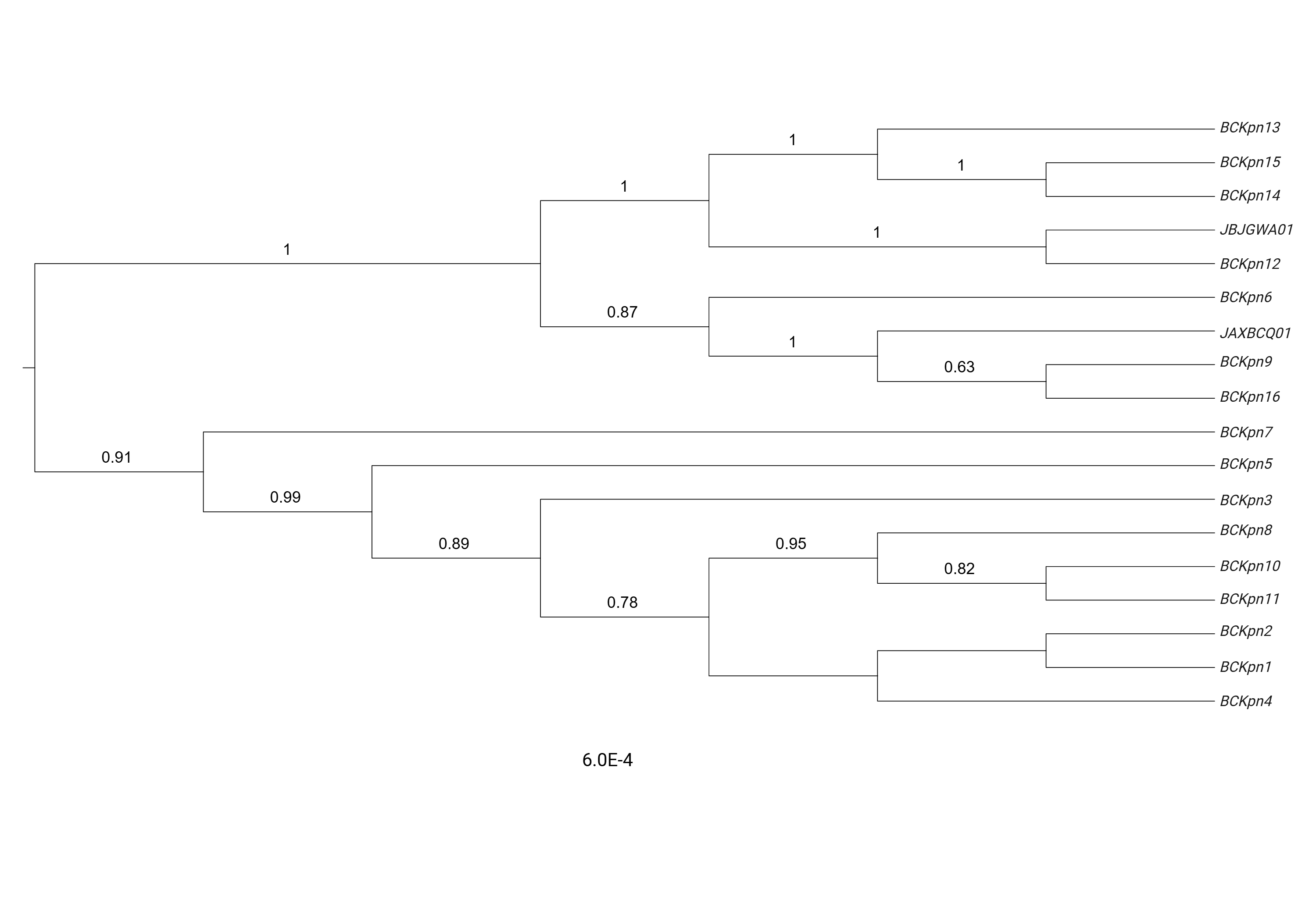

Supplement: Supplementary Figure 1 — High-resolution phylogenetic tree of KPC-Kp isolates with complete SH-like local support values at all nodes. [file Image_1.tiff]
